# Distinct Gene Expression Profiles in Viable Hepatocellular Carcinoma Treated With Liver-Directed Therapy

**DOI:** 10.3389/fonc.2022.809860

**Published:** 2022-06-17

**Authors:** Kelley G. Núñez, Tyler Sandow, Meredith A. Lakey, Daniel Fort, Ari J. Cohen, Paul T. Thevenot

**Affiliations:** ^1^ Institute of Translational Research, Ochsner Health System, New Orleans, LA, United States; ^2^ Interventional Radiology, Ochsner Health System, New Orleans, LA, United States; ^3^ Ochsner Biorepository, Ochsner Health System, New Orleans, LA, United States; ^4^ Centers for Outcomes and Health Services Research, Ochsner Health System, New Orleans, LA, United States; ^5^ Multi-Organ Transplant Institute, Ochsner Health System, New Orleans, LA, United States; ^6^ Faculty of Medicine, University of Queensland, Brisbane, QLD, Australia

**Keywords:** tumor microenvironment, transcriptomics, liver transplantation, intrahepatic spread, immune infiltration

## Abstract

**Background:**

Hepatocellular carcinoma is a heterogeneous tumor that accumulates a mutational burden and dysregulated signaling pathways that differ from early to advanced stages. Liver transplant candidates with early-stage hepatocellular carcinoma (HCC) undergo liver-directed therapy (LDT) to delay disease progression and serve as a bridge to liver transplantation (LT). Unfortunately, >80% of LDT-treated patients have viable HCC in the explant liver, dramatically increasing recurrence risk. Understanding the effect of LDT on early-stage HCC could help identify therapeutic targets to promote complete pathologic necrosis and improve recurrence-free survival. In this study, transcriptomic data from viable HCC in LDT-treated bridged to transplant patients were investigated to understand how treatment may affect tumor signaling pathways.

**Methods:**

Multiplex transcriptomic gene analysis was performed with mRNA extracted from viable tumors of HCC patients bridged to transplant using LDT. The NanoString nCounter^®^ Tumor Signaling 360 panel was used that contained 780 genes from 48 pathways involved in tumor biology within the microenvironment as well as antitumoral immune responses.

**Results:**

Hierarchical clustering separated tumors into three subtypes (HCC-1, HCC-2, and HCC-3) each with distinct differences in anti-tumoral signaling and immune infiltration within the tumor microenvironment. Immune infiltration (neutrophils, T cells, and macrophages) were all lowest in subtype HCC-3. The tumor inflammatory signature consisting of 18 genes associated with PD-1/PD-L1 inhibition, antigen presentation, chemokine secretion, and adaptive immune responses was highest in subtype HCC-1 and lowest in HCC-3. History of decompensation and etiology were associated with HCC subtype favoring downregulations in inflammation and immune infiltration with upregulation of lipid metabolism. Gene expression among intrahepatic lesions was remarkably similar with >85% of genes expressed in both lesions. Genes differentially expressed (<8 genes per patient) in multifocal disease were all upregulated in LDT-treated tumors from pathways involving epithelial mesenchymal transition, extracellular matrix remodeling, and/or inflammation potentially implicating intrahepatic metastases.

**Conclusion:**

Incomplete response to LDT may drive expression patterns that inhibit an effective anti-tumoral response through immune exclusion and induce intrahepatic spread.

## Introduction

Hepatocellular carcinoma (HCC) is the fourth leading cause of cancer-related death in the world (1). Most HCC develops in the background of cirrhosis caused by viral hepatitis, nonalcoholic, or alcoholic steatohepatitis. The mutational landscape of HCC is driven by etiology with the mutational burden increasing as the HCC progresses. Key driver mutations have identified several mutational signatures based on etiology (2–4). An initial study on the mutational landscape of resected HCC revealed 6 distinct groups (G1-G6) based on genetic, clinical, and pathological characteristics (5). Additional studies were conducted to pinpoint the driver mutations within HCC but focused on resected HCC (2, 3) lacking the complexities of cirrhosis. A large- scale study combining both genomic and transcriptomic profiling on early to advanced stages of HCC with cirrhosis revealed links between specific mutations and clinicopathological phenotypes with a 5-gene score predictive of overall survival in resected HCC (6). Transcriptomic data on HCC have also shown differences in immune infiltration that are associated with recurrence risk (7). While our understanding of the transcriptional landscape of very early and advanced HCC has improved, early-stage HCC remains incompletely characterized, particularly with regard to the impact of liver-directed therapy.

Liver transplantation remains the only option for cure of non-resectable early-stage HCC with underlying cirrhosis. Liver-directed therapies (LDT) are used to maintain tumor burden and the bridge to transplantation. Radiographic response to LDT has provided insight into the tumor biology of HCC. Patients with non-objective responses to LDT have a shorter intention to treat survival (8) and an increased risk of recurrence post-transplant (9). However, despite achieving complete radiographic responses, 25-43% of patients require additional treatment (8, 10) while >80% of treated tumors remain viable on explant (8). Failure to respond to LDT and the presence of viable tumors increase the risk of post-transplant recurrence (9, 11, 12) with dismal outcomes.

LDT targets HCC directly through ablative, chemo-, or radioembolization to kill cancer cells and promote an anti-tumoral response. Treatment with drug-eluting embolic transarterial chemoembolization (DEE-TACE), one type of LDT, causes hypoxia and unloads the chemotherapeutic doxorubicin, to induce apoptosis while promoting immunogenic cell death (13). LDT has been shown to cause an immune response both systemically and within the tumor microenvironment (14–16) with release of tumor-specific antigens to aid in recognition (15, 17). The liver itself is tolerogenic and, combined with an immunosuppressive tumor microenvironment, may inhibit an effective anti-tumoral response. LDT-induced molecular changes within HCC and the impact on surrounding tumor microenvironments remain understudied.

In this study, activated tumor signaling pathways were investigated in HCC patients bridged to transplantation with LDT. Our approach was to investigate mRNA expression profiles of early-stage HCC patients treated with DEE-TACE with viable tumors on explant. This approach allows for the characterization of LDT-resistant HCC and the impact of treatment on HCC signaling pathways.

## Methods and Materials

### Patient Cohort

HCC tissue was obtained from patients enrolled in an IRB-approved, observational study of patients receiving liver-directed therapy as a bridge to liver transplantation (Ochsner Health, protocol number 2016.131.B from 08/15/2016 – 12/15/2018). Criteria for the study included: patients diagnosed with HCC and scheduled to undergo LDT at Ochsner Health System. Exclusion criteria included: <18 years, absent HCC diagnosis, metastatic disease, and prescribed systemic therapy. Specimens were obtained from 17 patients with a total of 21 tumors available for analysis. An initial HCC diagnosis was made by board certified radiologists according to Liver Imaging – Reporting and Data System (LIRADS) criteria or biopsy-confirmed by a hepatobiliary pathologist according to the guidelines of the Organ Procurement and Transplant Network (1). Initial HCC staging was made according to the Barcelona-Clinic Liver Cancer (BCLC) system (18). After liver transplantation, explant tumor tissue was examined for histological grade, presence of lymphovascular invasion, pathological staging, and percentage necrosis by a board-certified pathologist (**Table 1**). The histological grade was determined based on Edmondson and Steiner system. Demographics, staging, tumor burden, and LDT history were extracted from the electronic medical record (**Table 1**). Decompensation status was determined based on the presence of ascites, hepatic encephalopathy, jaundice, and/or esophageal varices with bleeding that required medical intervention.

**Table 1:** Study cohort demographics.

Demographic	Cohort
**Patients, n (%)**	17 (100)
**Age at diagnosis, years (IQR)**	60 (58 - 65)
**Sex, male (%)**	13 (62)
**Cirrhotic etiology, n (%)**	
HCV	13 (76)
Other	4 (24)
**Scores and Staging at Diagnosis**	
**ECOG Performance, n (%)**	
0	14 (82)
1	3 (18)
**Child-Pugh of A, n (%)**	
A	9 (53)
B	8 (47)
**BCLC Stage, n (%)**	
A	16 (94)
B	1 (6)
**Tumor Burden at Diagnosis**	
**Largest lesion, cm (IQR)**	2.9 (2.2 - 3.8)
**Milan, within criteria (%)**	19 (90)
**First-Line Liver-Directed Therapy**	
**DEE-TACE, n (%)**	17 (100)
**Treatment Response to First-Line LDT**	
**Complete, n (%)**	6 (38)
**Partial/Stable/Progressive, n (%)**	10 (62)
**Additional LDT prior to LTx**	
**Total number of LDT, median (IQR)**	3.0 (2.0 - 4.5)
**Explant Pathology**	
**Histological Grade, n (%)**	
Grade 1	6 (29)
Grade 2	11 (52)
Grade 3	4 (19)
**Tumor Focality, n (%)**	
Solitary	10 (59)
Multifocal	7 (41)
**Lymphovascular Invasion, n (%)**	
Yes	1 (5)
**Pathological Staging, n (%)**	
T1	4 (19)
T2	17 (81)

IQR, Interquartile range; HCV, Hepatitis C virus; NASH, Nonalcoholic steatohepatitis; ASH, Alcoholic steatohepatitis; ECOG, Eastern Cooperative Oncology Group; BCLC, Barcelona Clinic Liver Cancer; LTx, Liver transplantation; AFP, alpha-fetoprotein; DEE-TACE, Doxorubicin-eluting embolic transarterial chemoembolization.

### Liver-Directed Therapy and Bridge to Liver Transplantation

The recommendation to receive liver-directed therapy as a bridge to liver transplantation was made by a multi-disciplinary board consisting of interventional radiologists, hepatologists, and transplant surgeons. Eligible patients for LDT met the following criteria: LIRADS or biopsy-confirmed HCC, BCLC A or B, Eastern Cooperative Oncology Group score 0-1, Child Pugh score A-B, and no evidence of co-malignancy. All patients in the analysis cohort received drug-eluting embolic transarterial embolization (DEE-TACE) utilizing doxorubicin. Selection of DEE-TACE was based on performance status, tumor characteristics, and stage of disease. All patients in the institution’s bridge to liver transplantation protocol underwent liver-directed therapy. During the study duration, DEE-TACE was the predominate LDT at our institution (08/15/2016 – 12/15/2018). All DEE-TACE procedures were technically successful with delivered doses ranging from 10-50 mg of doxorubicin per treatment. Follow-up imaging using triple-phase computerized tomography or magnetic resonance was performed approximately 30 days post-procedure. Response to LDT was evaluated by board certified interventional radiologist (>5 years’ experience) using the Response Evaluation Criteria in Solid Tumors modified for HCC (mRECIST) (2). Patients with residual viable HCC post-LDT received additional treatment cycles until complete tumor response. Patients with a complete radiographic response were monitored for recurrence every 3 months until liver transplantation. Pre-surgical response was determined by last imaging available prior to liver transplant. Treatment history for each lesion was tracked through interventional radiology reports that recorded targeted lesions by segment. Incidental findings of HCC in the pathology report were documented to the segment and cross-referenced with LDT treatment history.

### Gene Expression Profile

Five μm thick scrolls were cut from formalin-fixed, paraffin-embedded tumor tissue blocks. Total mRNA was extracted using AllPrep DNA/RNA FFPE kit (Qiagen, Germantown, MD) following manufacturer’s protocol. RNA integrity (via 260/280 ratio) and quantification was determined using Nanodrop (ThermoFisher, Waltham, MA). The Tumor Signaling 360 panel™ (NanoString Technologies, Seattle, Washington) was used to characterize mRNA expression profiles in 780 genes involved in tumor signaling and immune infiltration within the tumor microenvironment. The tumor signaling panel consisted of 48 gene sets within 10 core themes (**Supplemental Table 1**). Data was acquired using nCounter™ (NanoString Technologies, Seattle, WA). Raw data was analyzed using nSolver™ version 4.0 (NanoString Technologies, Seattle, WA) with background threshold set at counts 25 based on the maximum average count plus 3 standard deviations of the negative control probes. Raw data was then normalized using the geometric mean of positive probes selected using default settings within nSolver™ which utilizes the geNorm algorithm (19). In each sample, genes were excluded if not above the sample threshold. Heatmap on normalized data plotted by Z-score was generated using nSolver™. Genes present in the heatmap, that were below threshold, were excluded from downstream analysis. Directed global significance scores used annotations based on Gene Ontology, Reactome, and KEGG and were calculated according to algorithm in nSolver (https://www.nanostring.com/wp-content/uploads/2020/12/MAN-10030-03_nCounter_Advanced_Analysis_2.0_User_Manual.pdf) (20).

### Histology

Paraffin-embedded tumor tissue blocks were cut into five um thick sections and stained with hematoxylin and eosin (H&E).

### T Cell Phenotyping

Peripheral blood was collected prior to liver transplantation using BD Vacutainer^®^ cell preparation tubes with sodium citrate (BD Biosciences) and processed according to manufacturer’s protocol. Peripheral blood mononucleated cells were isolated, suspended in cryopreservation media, and stored at -80˚C until analysis. Naïve, memory, exhausted, and senescent T cell phenotyping panels were used to characterize population frequencies. All four panels contained CD3, CD56, CD4, and CD8 markers with 3 unique markers to define each T cell subpopulation. T cell panels were defined as follows: naïve (CCR7, CD62L, and CD45RA), memory (CCR7, CD69, and CD45RO), senescent (KLRG1, CD57, and CD28), and exhausted (CTLA4, LAG3, and PD-1).

### Statistical Analysis

All data analysis was performed in JMP 13.0 (SAS Institute Inc.). All graphical output was generated using GraphPad Prism 8.0 (GraphPad Software Inc.). Continuous data was expressed as median with interquartile (IQR) range. The non-parametric test, Wilcoxon, was used to test the association between categorical and continuous data. Differentially expressed genes between two samples using normalized data with log2 fold changes >2.0 were deemed as statistically significant. Two-way ANOVA with multiple comparisons were performed for statistical analysis of grouped data.

### Transcriptomic Statistical Analysis

Direct global significance scores were determined using normalized data for each gene in each pathway with default settings. The pathway scores from each subtype were then compared using an ANOVA to test for significance with normalized data. The differential expression module was used to determine which genes were significantly differentially expressed between two samples. P-values were adjusted using the moderately conservative Benjamini-Yekutieli method for false discovery rates with significance of <0.05. Total coverage of panel was determined by removing positive and negative controls and counting genes above background threshold including housekeeping genes for a total of 780 genes. Infiltrating immune cell type scores were determined using nSolver™ with default settings based on normalized mRNA counts from genes specific to each cell type as used previously (20). The log2 counts were then subtracted from the total tumor infiltrating lymphocyte score which combined the average score from all immune cell types.

## Results

### Distinct Gene Signatures in Viable Hepatocellular Carcinoma From Explant

Activated signaling pathways in viable HCC treated with LDT were investigated using an mRNA expression panel (780 genes) spanning genes involved in tumorigenesis, metastasis, and immune response. Men comprised 62% of the study cohort with most patients having hepatitis C virus as the primary cirrhosis etiology. The cohort was 94% (16/17 patients) Barcelona Clinic Liver Cancer Stage A with patients receiving a median of 3 LDT treatments prior to liver transplantation (**Table 1**). A total of 21 HCC viable tumors from 17 patients were available from explant containing a mixture of LDT-treated (n=13) and treatment-naïve tumors (n=8). Of the 780 genes probed, 58% (451/780) were present in all 21 samples including all 20 housekeeping genes, while <1% (5/780) of panel genes were completely absent (**Supplemental Table 2**). Ten genes were excluded due to expression below background in each individual sample. The most highly expressed genes (21/25, 84%) were grouped in core themes of tumor-promoting inflammation, avoiding immune destruction, or deregulating cellular energetics (**Supplemental Table 3**).

**Figure 1:**
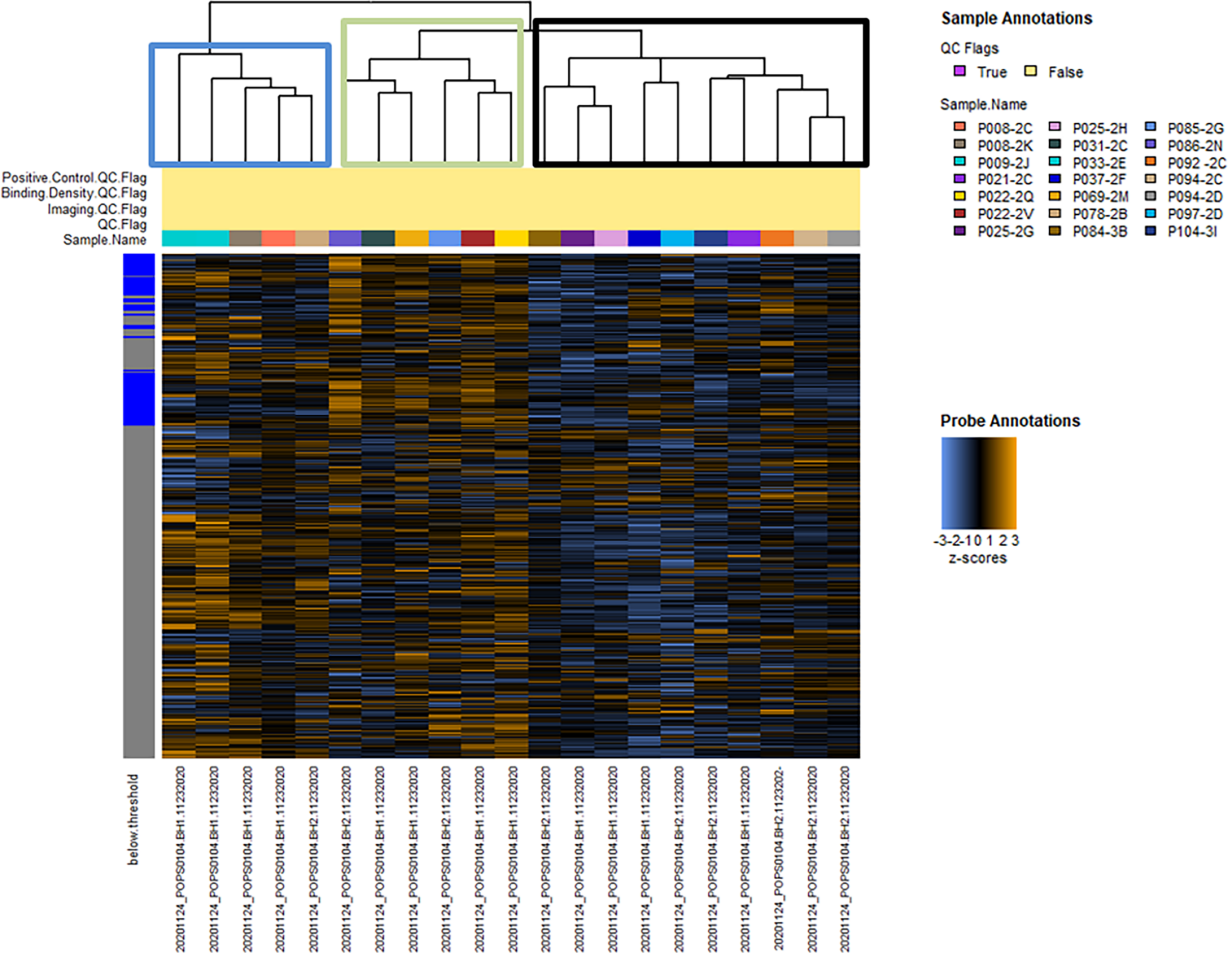
Hierarchical clustering of normalized data between 21 hepatocellular carcinoma tumors from 17 patients. Hierarchical clustering of 760 tumor signaling genes. No samples were flagged for quality control. Heatmap colors: blue indicates low expression, black indicates average, and orange indicates high expression. Tumors clustered into three nodes (HCC-1 - blue, HCC-2 - green, and HCC-3 - black boxes).

Hierarchical clustering of signaling pathway genes separated tumors into three distinct nodes (HCC-1, HCC-2, and HCC-3) (**Figure 1**). Directed global significance scores were used to determine which signaling pathways were up or downregulated between subtypes in combination (**Table 2**). There was a shift towards decreased expression of genes within pathways for inflammation, metastasis, and immune evasion between the subtypes. Genes involved in lipid metabolism were highly expressed in HCC-3 compared to HCC-1 and HCC-2. A total of 92 genes were differentially expressed between HCC-1 and HCC-3 subtypes with 9 genes upregulated and 83 genes downregulated (**Supplemental Table 2**). Further characterization of the top up and downregulated genes found in HCC-3 showed a shift towards downregulating genes involved in antigen-presentation, inflammation, and tumor suppressor genes, with upregulations in genes involved in oxidative stress responses and metabolism (**Table 3**).

**Table 2:** Directed global significance scores of top 20 differentially expressed pathways.

Core Theme	Pathway	HCC-2 vs HCC-1 GS Score	HCC-3 vs HCC-2 GS Score	HCC-3 vs HCC-1 GS Score	P Value*
Tumor-promoting Inflammation	Inflammation	-2.23	-1.70	-3.37	0.002
	Chemokine Signaling	-1.81	-2.18	-3.21	0.002
	Interleukin Signaling	-2.05	-1.00	-2.66	0.005
	JAK-STAT Signaling	-1.70	-1.48	-2.57	0.003
	Interferon Response	-1.46	-1.42	-2.42	0.332
	NF-κB Signaling	-1.24	+1.48	-2.29	0.001
Sustaining Proliferative Signaling	Notch Signaling	-2.25	-1.68	-3.75	0.001
	TGF-β Signaling	-2.20	-1.72	-3.59	0.002
	FGFR Signaling	-1.22	-1.08	-2.19	0.002
	Wnt Signaling	-1.74	+0.50	-2.05	0.003
	MET Signaling	-1.39	-1.05	-2.05	0.002
Activating Invasion and Metastasis	ECM Remodeling & Metastasis	-2.42	-1.49	-3.69	0.002
	Cell Adhesion & Motility	-2.12	-1.34	-3.19	0.002
	Hippo Signaling	-1.49	-1.39	-2.65	0.002
	EMT	-1.50	-1.06	-2.28	<0.001
Avoiding Immune Destruction	Cytotoxicity	-2.35	-1.60	-3.02	0.003
	TCR Signaling	-2.19	-1.06	-2.85	0.003
	T-cell Exhaustion	-2.94	+0.81	-2.81	0.006
	Myeloid Immune Evasion	-1.58	-1.54	-2.58	0.003
Inducing Angiogenesis	PDGF Signaling	-2.08	-1.09	-2.84	0.005
	HIF1 Signaling	-2.11	-1.19	-2.84	<0.001
	VEGF Signaling	-1.74	-1.22	-2.19	0.003
Deregulating Cellular Energetics	Lipid Metabolism	0.57	+3.87	+3.35	0.003

Global Significance Score (GS Score); Extracellular Matrix (ECM); Transforming Growth factor-beta (TGF-β); T cell Receptor (TCR); Platelet-Derived Groth Factor (PDGF); Hypoxia-Inducible Factor-1 (HIF1); Janus Kinase-Signal Transducer (JAK-STAT); Nuclear Factor Kappa-Light-Chain-Enhancer of Activated B cells (NF-κB); Epithelial Mesenchymal Transition (EMT); Vascular Endothelial Growth Factor (VEGF); Fibroblast Growth Factor Receptor (FGFR). Z-Score is score for first designed number. *P value based on comparsions of pathway scores between all three subtypes.

**Table 3:** Top up and downregulated genes differentially expressed in HCC-3 subtype.

Upregulated				
Gene	Core Theme(s)	Pathway	Log2 Fold Change in HCC-3	P-Value
*CYP4A11/22*	Deregulating Cellular Energetics	Lipid Metabolism	+1.83	>0.001
*AR*	Sustaining Proliferative Signaling, Activating Invasion and Metastasis	Androgen Signaling, EMT	+1.65	0.001
*IDH1*	Deregulating Cellular Energetics	Glucose Metabolism, mTOR Signaling	+1.48	>0.001
*ESRP2*	Sustaining Proliferative Signaling	FGFR Signaling	+1.32	>0.001
*MET*	Activating Invasion and Metastasis, Sustaining Proliferative Signaling	EMT, MAPK Signaling, MET Signaling	+1.25	>0.001
*SOD1*	Genome Instability & Mutation, Deregulating Cellular Energetics	DNA Damage Repair, Nrf2 & Oxidative Stress	+1.2	>0.001
*ATOX1*	Deregulating Cellular Energetics	Nrf2 & Oxidative Stress	+1.17	0.001
*TXN*	Deregulating Cellular Energetics	Nrf2 & Oxidative Stress	+1.11	>0.001
*LAMTOR2*	Sustaining Proliferative Signaling, Deregulating Cellular Energetics	MAPK Signaling, mTOR Signaling	+1.06	>0.001
**Downregulated**				
*HLADRB1*	Avoidign Immune Destruction, Tumor-Promoting Inflammation	Antigen Presentation, Interferon Response	-3.21	0.007
*NBL1*	Sustaining Proliferative Signaling	TGF-β Signaling	-3.21	0.046
*FGF7*	Sustaining Proliferative Signaling	FGFR Signaling, MAPK Signaling	-3.16	0.046
*LOX*	Activating Invasion and Metastasis, Sustaining Proliferative Signaling	EMT, TGF-β Signaling	-3.08	0.035
*LAIR1*	Tumor-Promoting Inflammation	Inflammation	-2.81	0.047
*SPINT1*	Genome Instability & Mutation, Sustaining Proliferative Signaling	Epigenetic & Transcriptional Regulation, MET Signaling	-2.77	0.046
*PLAU*	Tumor-Promoting Inflammation	Inflammation, NF-κB Signaling	-2.75	0.046
*NCF2*	Deregulating Cellular Energetics	Nrf2 & Oxidative Stress	-2.60	0.048
*ITGB8*	Activating Invasion and Metastasis	Cell Adhesion & Motility, ECM Remodeling & Metastasis	-2.56	0.028
*GPNMB*	Avoiding Immune Destruction	Tumor Antigen	-2.54	0.022

Transforming Growth factor-beta (TGF-β); Fibroblast Growth Factor Receptor (FGFR); Epithelial Mesenchymal Transition (EMT); Nuclear Factor Kappa-Light-Chain-Enhancer of Activated B cells (NF-kB); Extracellular Matrix (ECM); Platelet-Derived Groth Factor (PDGF); Hypoxia-Inducible Factor-1 (HIF1); Vascular Endothelial Growth Factor (VEGF).

### Tumor Microenvironment Immune Cell Infiltration and Inflammation for Each HCC Subtype

**Figure 2:**
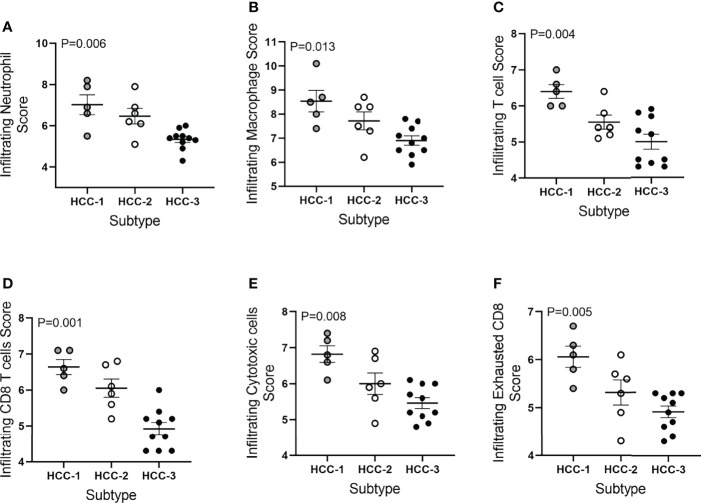
Immune cell type profiling score of HCC tumors based on subtype. Infiltrating immune cell type scores included **(A)** neutrophils, **(B)** macrophages, **(C)** T cells, **(D)** CD8 T cells, **(E)** cytotoxic cells, and **(F)** exhausted CD8 T cells. Abundance was determined using log2 normalized mRNA counts for each specific cell type marker and subtracting the total TIL score (average B cell, T cell, CD45, macrophage, and cytotoxic cell scores).

**Figure 3:**
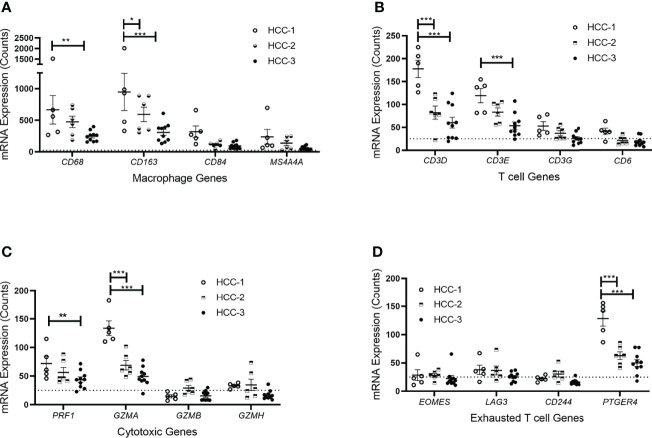
Immune cell type-related gene expression of HCC tumors based on subtype. mRNA expression counts for genes within each cell type including **(A)** macrophages, **(B)** T cells, **(C)** cytotoxic T cells, and **(D)** exhausted T cells grouped by subtype. Dotted line represents gene expression counts below background thresholds. *P < 0.05, **P < 0.01, ***P < 0.001.

Tumor infiltrating immune cell populations were evaluated in viable HCC after liver transplantation using the immune cell type profiling score which quantifies the abundance of immune cell populations based on cell-specific marker genes. The trend of downregulating genes involved in antigen-presentation and inflammation corresponded with a decrease in the abundance of infiltrating immune cell populations. The subtype HCC-3 had the fewest neutrophils, cytotoxic cells, macrophages, and several T cell populations (**Figure 2**). Hematoxylin and eosin staining showed infiltrating immune cells varied between nodes (**Supplemental Table 4**). Expression of immune cell type-related genes were examined within each HCC subtype. Markers specific for macrophages (CD68 and CD163) had decreased expression between HCC-1 and HCC-3 while no difference in expression was observed in the M2 macrophage marker MS4A4A (**Figure 3A**). For T cell-specific genes, CD3 delta and epsilon expression were lowest in HCC-3 subtype while CD3 gamma chain expression remained unchanged (**Figure 3B**). Observed decreases in cytotoxic cells between subtypes paralleled the decreasing trend in perforin 1 and granzyme A mRNA (PRF1 and GZMA) expression (**Figure 3C**). Though some lymphocytes express both granzyme A and B, expression of granzyme B was below background for all HCC subtypes regardless of infiltrating cytotoxic cell abundance. Despite abundant differences in exhausted CD8 T cells, only prostaglandin E receptor 4 (PTGER4) mRNA was observed to be differentially expressed between the HCC subtypes (**Figure 3D**).

Next, the tumor inflammatory signature that consisted of 18 genes associated with PD-1/PD-L1 inhibition, antigen presentation, chemokine secretion, and adaptive immune responses was assessed for each HCC subtype (**Table 4**). HCC-1 consistently showed higher levels of proinflammatory genes within the tumor microenvironment with a trend toward downregulation in HCC-3 subtype. Gene expression of both CCL5 and CXCR6, chemokines involved in T cell chemotaxis, along with CD8A and proteins involved in antigen presentation (HLA-DRA1 and HLA-DRB1) were all significantly downregulated in HCC-3 tumors.

**Table 4:** Tumor inflammation signature by subtype.

Gene	HCC-1	HCC-2	HCC-3	P-Value
***CCL5*, mean (IQR)**	678 (536 - 830)	387 (162 - 1112)	212 (113 - 300)	**0.011**
***CXCL9*, mean (IQR)**	77 (69 - 451)	296 (55 - 421)	94 (64 - 128)	0.439
***CD27*, mean (IQR)**	410 (240 - 547)	526 (265 - 720)	328 (274 - 425)	0.693
***CD8A*, mean (IQR)**	202 (128 - 276)	132 (63 - 239)	66 (28 - 80)	**0.008**
***CXCR6*, mean (IQR)**	82 (76 - 118)	47 (29 - 88)	27 (20 - 45)	**0.008**
***IDO1*, mean (IQR)**	23 (17 - 49)	40 (31 - 49)	28 (16 - 37)	0.321
***STAT1*, mean (IQR)**	661 (394 - 983)	716 (344 - 1326)	416 (288 - 1013)	0.699
***TIGIT*, mean (IQR)**	87 (54 - 99)	42 (22 - 48)	24 (16 - 40)	**0.020**
***LAG3*, mean (IQR)**	35 (24 - 53)	31 (24 - 49)	25 (17 - 32)	0.183
***PD-L2/PDCD1LG2*, mean (IQR)**	23 (17 - 46)	17 (12 - 26)	11 (7.0 - 18)	0.052
***PD-L1/CD274*, mean (IQR)**	29 (25 - 34)	42 (27 - 61)	30 (22 - 31)	0.262
***CD276*, mean (IQR)**	410 (240 - 547)	526 (265 - 720)	328 (274 - 245)	0.693
***HLA-DQA1*, mean (IQR)**	575 (159 - 1675)	132 (9.0 - 443)	5.0 (2.4 - 105)	**0.038**
***HLA-E*, mean (IQR)**	2292 (1813 - 2689)	1507 (1267 - 3029)	1255 (997 - 1856)	0.058
***PSMB10*, mean (IQR)**	202 (168 - 262)	145 (122 - 169)	135 (113 - 181)	**0.037**
***HLA-DRB1*, mean (IQR)**	4262 (2005 - 6083)	1675 (373 - 2159)	373 (228 - 710)	**0.004**
***CMKLR1*, mean (IQR)**	85 (69 - 153)	83 (63 - 123)	39 (32 - 52)	**0.005**
***NKG7*, mean (IQR)**	267 (211 - 420)	218 (153 - 374)	111 (80 - 172)	**0.011**

Chemokine ligand 5 (CCL5); CXC motif chemokine ligand 9 (CXCL9); CXC motif chemokine receptor 6 (CXCR6); Indoleamine 2,3-dioxygenase 1 (IDO1); Signal transducer and activator of transcription 1 (STAT1); T cell immunoreceptor with Ig and ITIM domains (TIGIT); Lymphocyte activating 3 (LAG3); Programmed cell death 1 ligand 2 (PD-L2); Programmed cell death 1 ligand 1 (PD-L1); Major histocompatibility complex class II DQ alpha 1 (HLA-DQA1); Major histocompatibility complex class I, E (HLA-E); Proteasome subunit beta type-10 (PSMB10); Major histocompatibility complex class II DR beta 1 (HLA-DRB1); Chemerin chemokine-like receptor 1 (CMKLR1); Natural killer granule protein 7 (NKG7). Bold p-value indicates values <0.050.

**Pre-Transplant Clinical Variables and T Cell Phenotypes**

>A logistic regression was performed to determine if any pre-transplant clinical variables were associated with the HCC subtype (**Table 5**). Analysis revealed that cirrhosis etiology and a history of decompensation prior to liver transplantation were associated with the HCC subtype. HCC-1 consisted of only patients with compensated cirrhosis, while HCC-2 and -3 were characterized by a higher percentage of patients with a history of decompensation. All patients with alcoholic hepatitis or nonalcoholic steatohepatitis were clustered within the HCC-3 subtype while those with HCV were found within all three subtypes. Initial tumor burden, treatment frequency, or response to first-line LDT, were similar among HCC subtype.

**Table 5:** Pre-transplant clinical variables by HCC subtype.

	HCC-1	HCC-2	HCC-3	P-Value
**Number of lesions**	5	6	10	** **
**Demographic**				
**Patients, n (%)**	4	5	8	
**Age at diagnosis, years (IQR)**	62 (59 - 68)	60 (55 - 63)	62 (57 - 65)	0.743
**Sex, male (%)**	1 (25)	5 (83)	7 (70)	0.071
**Cirrhosis etiology, n (%)**				**0.010**
HCV	5 (100)	6 (100)	5 (50)	
Other	0 (0)	0 (0)	5 (50)	
**Decompensation Status Prior to LTx**				**0.004**
**Compensated, n (%)**	5 (100)	1 (17)	3 (30)	
**Decompensated, n (%)**	0 (0)	5 (83)	7 (70)	
**Clinical Hepatology Labs Prior to LTx**				
**Sodium, mM (IQR)**	138 (137 - 138)	137 (132 - 139)	137 (135 - 141)	0.831
**Creatinine, mg/dL (IQR)**	0.8 (0.7 - 0.9)	0.9 (0.9 - 1.2)	1.0 (0.8 - 1.5)	0.111
**Bilirubin, mg/dL (IQR)**	1.9 (0.6 - 3.1)	3.0 (1.7 - 3.9)	1.1 (0.7 - 1.5)	**0.050**
**Albumin, g/dL (IQR)**	3.0 (2.4 - 3.0)	3.0 (2.4 - 3.4)	3.4 (2.6 - 3.7)	0.252
**INR, ratio (IQR)**	1.2 (1.0 - 1.6)	1.2 (1.2 - 1.3)	1.1 (1.0 - 1.3)	0.445
**MELD-Na, score (IQR)**	12 (9 - 17)	15 (12 - 20)	10 (8 - 16)	0.354
**Clinical Blood Labs Prior to LTx**				
**WBC, 10^3^/μL (IQR)**	3.9 (2.2 - 7.2)	4.4 (3.0 - 6.9)	4.2 (3.4 - 5.9)	0.793
**PMN, 10^3^/μL (IQR)**	2.4 (0.9 - 4.5)	2.9 (2.0 - 5.1)	2.8 (1.6 - 3.9)	0.771
**ALC, 10^3^/μL (IQR)**	1.2 (0.8 - 2.0)	0.9 (0.5 - 1.1)	1.3 (0.8 - 2.0)	0.234
**Mono, 10^3^/μL (IQR)**	0.4 (0.3 - 0.6)	0.5 (0.4 - 0.7)	0.6 (0.4 - 0.7)	0.349
**Platelets, 10^3^/μL (IQR)**	71 (41 - 91)	82 (65 - 128)	109 (66 - 142)	0.237
**Tumor Burden and Biomarkers**				
**Largest lesion, cm (IQR)**	2.6 (1.6 - 4.4)	2.3 (2.1 - 2.6)	2.5 (2.1 - 4.0)	0.694
**Milan, within criteria (%)**	75	100	90	0.427
**AFP, ng/mL (IQR)**	19 (8.3 - 26)	19 (5.0 - 101)	23 (4.0 - 66)	0.933
**Liver-Directed Therapy**				0.380
**Number of Treated Lesions**	4 (75)	4 (67)	5 (50)	
**Treatment Response to First-Line LDT**				0.874
**Complete, n (%)**	1 (25)	2 (40)	2 (29)	
**Partial/Stable/Progressive, n (%)**	3 (75)	3 (60)	5 (71)	

LTx, Liver transplant; IQR, Interquartile range; INR, International normalized ratio; MELD-Na, Model of End stage Liver Disease – Sodium; WBC, White blood count; PMN, Polymorphonucleated leukocyte count; ALC, Absolute lymphocyte count; mono, Monocytes; AFP, alpha-fetoprotein; LDT, Liver-directed therapy. Bold p-value indicates values <0.050.

Lack of immune infiltration within the tumor microenvironment may be caused by T cell imbalance in the periphery. To investigate this, circulating T cell phenotypes prior to liver transplantation were determined using flow cytometry. While total T cells were not different among patients grouped by HCC subtype, circulating memory T cell differences were observed with the lowest abundance in patients grouped within the HCC-3 subtype (**Table 6**).

**Table 6:** Peripheral T cell phenotypes prior to liver transplant by subtype.

	HCC-1	HCC-2	HCC-3	P-Value
**T cell Type**				** **
**CD3% of total CD45; median (IQR)**	41 (39.3 - 47.9)	43.6 (32.2 - 50.4)	37.3 (33.2 - 41.0)	0.387
**Naïve T cells**	** **	** **	** **	** **
**Naïve CD3% of total CD3; median (IQR)**	2.8 (0.9 - 7.6)	3.1 (1.8 - 5.6)	2.5 (1.4 - 4.8)	0.886
**Memory T cells**				** **
**Memory CD3% of total CD3; median (IQR)**	10.9 (7.6 - 15.1)	15.9 (9.3 - 18.6)	5.7 (4.8 - 7.1)	**0.005**
**Effector T cells**				
**Effector CD3% of total CD3; median (IQR)**	29.4 (18.1 - 37.8)	33.5 (17.4 - 36.1)	22.5 (15.7 - 30.2)	0.512
**Senescent T cells**				** **
**Senescent CD3% of total CD3; median (IQR)**	4.6 (1.5 - 9.8)	2.2 (1.0 - 5.5)	1.0 (0.11 - 5.3)	0.495
**Exhausted T cell Phenotype**				
**CD3 PD-1, MFI; median (IQR)**	753 (737 - 764)	768 (745 - 848)	730 (703 - 770)	0.061
**CD3 LAG3, MFI; median (IQR)**	189 (187 - 192)	194 (190 - 196)	190 (189 - 193)	0.104
**CD3 CTLA4, MFI; median (IQR)**	355 (340 - 363)	358 (353 - 369)	356 (352 - 358)	0.212

PD-1, Programmed cell death protein-1; LAG3, Lymphocyte activating 3; CTLA4, Cytotoxic T-lymphocyte-associated protein 4. Bold p-value indicates values <0.050.

### Explant Pathology, Post-Transplant Recurrence, and HCC Subtype

Subtypes HCC-2 and HCC-3 were combined and explant pathology and recurrence rates were compared with HCC-1 (**Table 7**). Median time between LDT treatment and liver transplantation was 287 days (IQR: 201 - 657) While no differences in histological grade or staging were observed between the subtypes, one patient with lymphovascular invasion was found in the HCC-2 and -3 groups. In this study, three patients (18%) developed post-transplant recurrence. Recurrence for each patient occurred <2 years after liver transplantation with a median of 363 days post-transplant (IQR: 305 – 481). While no significant differences were observed in recurrence and the HCC subtype, two patients who recurred had the HCC-3 subtype.

**Table 7:** Explant characteristics and recurrence by subtype.

	Subtype		
	HCC-1	HCC-2 and HCC-3	P-Value
**Number of lesions, n**	5	16	** **
**Explant pathology**			
**Histological Grade, n (%)**			0.627
Well differentiated	1 (20)	5 (31)	
Moderately or poorly differentiated	4 (80)	11 (69)	
**Tumor Focality, n (%)**			0.525
Solitary	3 (60)	7 (44)	
Multifocal	2 (40)	9 (56)	
**Lymphovascular Invasion, n (%)**			0.567
Yes	0 (0)	1 (6)	
**Pathological Staging, n (%)**			0.951
T1	1 (20)	3 (19)	
T2	4 (80)	13 (81)	
**Recurrence, n (%)**			0.296
Yes	0 (0)	3 (19)	
No	5 (100)	13 (81)	

### Gene Expression in LDT-Treated Multifocal HCC

To investigate tumor signaling pathways within intrahepatic tumors, gene expression data from four patients with multifocal disease were compared. Each patient had an LDT-treated and treatment-naïve tumor. Multifocal tumors shared remarkable similarities with >85% expressed genes found in both tumors among studied patients. A total of 14 genes from all patients with multifocal disease were differentially expressed >5-fold in treated tumors from pathways involving epithelial mesenchymal transition, extracellular matrix remodeling, and/or inflammation (**Table 8**). These results suggest genes involved in intrahepatic spread are activated in partially treated lesions.

**Table 8:** Shared gene expression within multifocal HCC on explant.

Subtype	Tumor #	Genes Shared (n, coverage of panel)	Shared Genes Between Intrahepatic Tumors	Treated History of Lesion, (n)	Necrosis (%)	Pathways Upregulated >5 fold
HCC-1	1	587, 75%	91%	DEE-TACE (3)	0	EMT, Interleukin Signaling, Cell Adhesion and Motility, ECM Remodeling & Metastasis, Inflammation
HCC-1	2			None	0	None
HCC-2	1	446, 57%	85%	DEE-TACE (1)	10	EMT, ECM Remodeling & Metastasis, Glutamine
HCC-2	2			None	0	None
HCC-3	1	521, 67%	91%	DEE-TACE (2)	0	Inflammation
HCC-3	2			None	0	None
HCC-3	1	620, 79%	93%	DEE-TACE (3)	40	EMT, ECM Remodeling & Metastatsis
HCC-3	2			None	0	None

EMT, Epithelial Mesenchymal Transition; ECM, Extracellular Matrix.

## Discussion

HCC is a heterogenous tumor with a variety of mutations, dysregulated signaling pathways, and complex interactions within the tumor microenvironment. Over the last decade, there has been tremendous progress in uncovering signaling pathways critical for HCC progression (21–23). However, much of this work has focused on pathways dysregulated in cell lines (24–26) or resected tissue (4, 27, 28), either lacking the complexities of a tumor microenvironment and background cirrhosis. Studies in non-cirrhotic, resected HCC have identified several HCC subtypes based on genetic mutations, pathology, and transcriptomics data (4, 7, 29–31) [Hoshida, 2009 (29) #14] (30). While this research has provided insight into driver mutations and the immune landscape within the TME of HCC, the impact of LDT on tumor signaling pathways in cirrhotic patients are not completely understood.

With increased adherence to HCC surveillance programs, the rates of early-stage diagnosis have increased allowing access to treatments with curative intent such as liver transplantation. LDT treatments for HCC have also increased in usage over the years. While DEE-TACE causes immunogenic cell death through doxorubicin, any treatment-induced molecular changes within the tumor may facilitate more aggressive biology. In this study, transcriptomic profiles of post-LDT viable HCC revealed distinct subtypes that differed in expression of inflammation, immune evasion, angiogenesis, and sustaining proliferation signaling pathways. Several common dysregulated pathways for HCC such as VEGF (32), MAPK (28), AKT/mTOR (33), β-catenin/Wnt (34), JAK/STAT (34), and Notch (35) were also activated in viable HCC regardless of treatment history.

The goal of LDT is not only to kill cancer cells but also release tumor-specific antigens to promote inflammation and elicit an adaptive immune response. The presence and degree of antitumoral immune response is dependent on the TME. In this study, HCC-3 subtype had downregulation of both inflammation-related and antigen-presenting genes, with decreased immune cell infiltration suggesting immune evasion. In line with our results, studies have shown decreased intra-tumoral T cells in HCC (36, 37). TACE has been shown to decrease T cell infiltration regardless of treatment response (38) although the mechanisms remain unclear. Within the HCC-3 cohort, 50% of tumors were treatment naïve with 4 out of 5 patients having a treated tumor with complete pathological necrosis. The HCC-3 subtype was also characterized by downregulation of genes involved in T cell chemotaxis (*CCL5*) and cytotoxic proteins (perforin 1 and granzyme A) responsible for effector T cell and natural killer cell cytolysis. In addition, HCC-3 had significant downregulation of major histocompatibility complex class I and II components critical for effective antigen presentation. These data suggest the HCC-3 subtype is characterized by poor immune infiltration and impaired effector responses which could impact immune-mediated mechanisms of tumor cell death post-LDT, resulting in viable lesions at explant and increasing the risk of HCC recurrence post-transplant.

HCC transcriptomic profiles were associated with etiology of cirrhosis and history of decompensation but not radiographic measures of tumor burden. Most HCC develops on a background of cirrhosis, with etiology-specific mutation profiles (4). In this study, HCV-HCC tumors were evenly spread among each subtype while all non-HCV etiologies (NASH and ALD) clustered within HCC-3. Genes involved in lipid metabolism were the only pathway upregulated in HCC-3 compared to other subtypes. One of the hallmarks of NASH is the accumulation of macro- and microsteatosis with metabolic dysfunction (39). Ethanol has been shown to induce steatosis by promoting lipogenesis (40). Although only 2/8 (25%) of patients in the HCC-3 subtype had NASH, 6/8 (75%) had varying levels of macrosteatosis in the native liver at the time of transplantation. This could explain the upregulation in genes controlling lipid metabolism in HCC-3. NASH-HCC has also been shown to have decreased expression of genes involved in the Wnt signaling pathway (41) which could account for the downregulation of this pathway observed in the HCC-3 subtype.

Patients who experienced a decompensating event that required medical intervention were found in only the HCC-2 and HCC-3 subtypes. Cirrhosis-associated immune dysfunction (CAID) refers to the cyclic state of systemic inflammation and immunodeficiency that develops and worsens during the progression of compensated to decompensated cirrhosis [see review (42)]. In decompensated cirrhosis, CAID leads to impaired neutrophil (43) and monocyte (44) function with decreased proliferation and cytotoxic activity of both T cells and natural killer cells (42). While decompensation was not associated with cirrhosis etiology, the frequency of decompensation was highest in the HCC-2 and HCC-3 subtypes characterized by immune exclusion and possibly an immunodeficiency TME and warrants further investigation.

Well established prognostic factors for early recurrence of HCC after liver transplantation include vascular invasion, pre-transplant alpha-fetoprotein, viable lesions at explant, and response to LDT. In HCC patients bridged to transplantation with LDT, <90% of tumor necrosis was a risk factor for recurrence post-transplant (7, 45). Though we did not observe a significant difference in post-transplant recurrence and subtype, a higher recurrence rate in the HCC-3 subtype was observed, supporting that a phenotype of immune exclusion within the TME may impact post-transplant recurrence risk. Overall post-liver transplant recurrence rate for the cohort was 14%, equal to the center’s HCC recurrence rate and in agreement with literature (46).

Most HCC patients, who bridged to transplantation with LDT, underwent multiple treatments to maintain tumor burden within transplant criteria or to achieve cPN to treatment. Despite achieving complete radiographic responses to TACE, 43% of patients had recurrence of the treated tumor or developed a new tumor (10). In many cases, the new tumor failed to meet imaging diagnostic criteria and remained untreated at the time of liver transplantation. Treated and untreated HCC from the same patient were analyzed to determine whether the transcriptional profiles were similar. There was large degree of overlap (>85% of expressed genes shared) in multifocal tumors and upregulation of epithelial mesenchymal transition pathway in treated tumors. All treatment naïve tumors that failed to meet the HCC criteria prior to transplant were <1.8 cm in size, and were located adjacent to treated tumors. A recent comprehensive study found multifocal resected HCC had similar gene expression profiles in patients with intrahepatic metastases (47). cPN was not achieved and likely led to molecular signaling alterations that may have facilitated intrahepatic spread. Similar to this study, we observed similar transcriptional profiles in multifocal disease regardless of treatment history. This is in agreement with a recent transcriptomic immune profiling of TACE treated versus untreated HCC which found only a single differentially expressed gene among the two groups (38). A majority of the immune panel used in that study (90%) contained genes on immune responses and lacked an inflammation signature, while 70% the panel used in this study focused on tumor signaling pathways and included the inflammation signature. While DEE-TACE provides a means to maintain tumor burden and treat HCC, more research is warranted to determine if a viable tumor after treatment increases risk of intrahepatic metastases.

This study was a single center design and focused on only one liver-directed therapy modality. LDT outcomes are center-dependent and driven by the experience of the interventional radiologists performing the treatment. The transcriptomic profiles and gene expression overlap in multifocal disease may be dependent upon the DEE-TACE modality, which is predominantly a debulking therapy with limited capacity for treatment with curative intent. The transcriptional profiles obtained may be biased due to the selection of patients successfully bridged to liver transplantation. Patients with early-stage HCC progressing after LDT receive systemic therapies prior to tumor biopsy for mutational analysis. Therefore, we were unable to generate profiles for patients with tumor progression due to intermittent systemic therapy. The TACE Navigator Gene Signature was proposed for treatment prognosis (48). While this study was tested on multiple cohorts in treatment-naïve tumors in those that underwent resection and transplant, only a single gene (*UBB*) was present in the 780 gene panel used in this study which served as a housekeeping control. In our study, there was no relationship between tumor response and transcriptomic profile.

The data presented sheds light on the activated tumor signaling pathways in early-stage, non-resectable HCC after DEE-TACE treatment. Subtypes of HCC were determined based on mRNA expression profiles which included a subtype with decreased proinflammatory expression without abundant immune cell types in the TME. HCC which does not respond to a particular LDT modality are often treated with a different modality or, in the case of post-treatment tumor progression, combination LDT treatment with systemic agents. Recently, combination immunotherapy trials (EMERALD-1 and LEAP-012) target immune evasion mechanisms in order to increase objective response rates. Whether these combination therapies can circumvent immune evasion in LDT-resistant HCC is still unclear.

## Conclusion

This study highlights distinct signaling pathways in LDT-treated HCC bridged to liver transplantation. One subtype (HCC-3) was characterized by poor immune infiltration within the tumor microenvironment that could limit immune-mediated cell death after treatment and impact recurrence rates post-transplant. In addition, similar transcriptomic profiles were found within multifocal HCC patients with incompletely treated and treatment-naïve lesions suggestive of intrahepatic spread that warrants further investigation. In conclusion, understanding the pathways involved in early-stage HCC and the changes liver-directed therapies may induce will provide insight into potential combination therapies aimed at specific pathways to increase complete response rates in bridge to liver transplantation HCC population.

## Data Availability Statement

The original contributions presented in the study are included in the article/**Supplementary Materials**. Further inquiries can be directed to the corresponding author.

## Ethics Statement

The studies involving human participants were reviewed and approved by Ochnser Institutional Review Board. The patients/participants provided their written informed consent to participate in this study.

## Author Contributions

KN and PT designed the research. KN performed the experiments and analyzed the data. KN and PT wrote the manuscript. TS scored responses to treatment for all patients. DF was involved in statistical analysis for the study. ML provided immune scores from pathological analysis. KN, PT, and AC provided funding. AC and PT supervised the study. KN, AC, and PT revised the manuscript. All authors contributed to the research article and approved the submitted version.

## Funding

The authors declare that this study received funding from Nanostring Technologies. The funder was not involved in the study design, collection, analysis, interpretation of data, the writing of this article or the decision to submit it for publication.

## Conflict of Interest

The authors declare that the research was conducted in the absence of any commercial or financial relationships that could be construed as a potential conflict of interest.

## Publisher’s Note

All claims expressed in this article are solely those of the authors and do not necessarily represent those of their affiliated organizations, or those of the publisher, the editors and the reviewers. Any product that may be evaluated in this article, or claim that may be made by its manufacturer, is not guaranteed or endorsed by the publisher.
