# Supplementary figures and images for: Distinct Gene Expression Profiles in Viable Hepatocellular Carcinoma Treated With Liver-Directed Therapy
Source: Front Oncol. 2022 Jun 17;12:809860. doi: 10.3389/fonc.2022.809860 (PMC9248864; doi:10.3389/fonc.2022.809860)

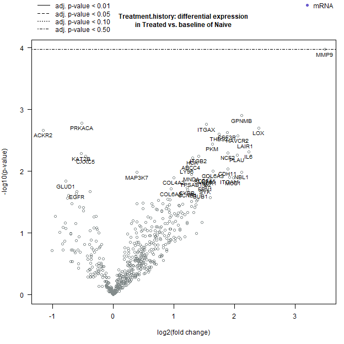

Supplement: Supplementary file 1 [file Image_1.tif]
